# The Maintenance of Health

**Published:** 1875-05-15

**Authors:** 


THE MAINTENANCE OF HEALTH. A medical work for lay readers. By J. Milner Fothergill, M.D. G. P. Putnam's Sons, New York.

This new work by Dr. Fothergill will no doubt be favorably received by its numerous readers, containing, as it does, much valuable information for the laity as well as the profession. Though much of its contents has been already published, the ideas are so arranged and brought out by the author as to impress the reader with their value, and it is doubtful whether any one can read the book without gathering much that will be useful to them.

The work is divided into several chapters, in the first of which the author tells how to maintain the health in youth, showing why some children are of a healthy, vigorous constitution while others are always sickly and puny; giving directions as to the food, clothing and exercise necessary to maintain a degree of health at this age. The second chapter is devoted to the changes in maturity, how the different habits affect the system, the occupations chosen for a livelihood, and their influence on the duration of life. And in the third chapter, when maturity has given place to advanced age, and the period of decay comes on, he informs the reader how the action of each of the bodily organs is best facilitated so as to still preserve the health. Besides these three chapters the book contains several others in which such subjects as food, clothes, stimulants and tobacco, and the effects of inheritance, are fully discussed. Not a little attention has been paid by the author to the subject of hygiene, and to the ill effects of mental overwork which is now so common among students and business men. In conclusion, he recommends certain things to be done in case of any of the emergencies of every-day life.

On the whole, it is just such a work as a great mass of the people need to instruct them how to preserve their moral and physical health.

